# Optimized Metabolic Conditions Reveal the Adipogenic Competence of Wharton’s Jelly MSCs: Insight from Literature Review and Experimental Validation

**DOI:** 10.1007/s12015-026-11196-4

**Published:** 2026-07-23

**Authors:** Cécile Pinault, Audrey Cras, Michel-Gabriel Cazenave, Anne Schnitzler, Jeanne Rancic, Jerome Larghero, Sophie Vacher, Curtis L Cetrulo, Daniel Balvay, Ivan Bieche, Alexandre G. Lellouch, David M. Smadja

**Affiliations:** 1 Paris Cité University, INSERM PARCC, Paris, 75015 France; 2 AP-HP, Cell therapy department, INSERM Centre d’Investigation Clinique en Biothérapies CIC-BT, Saint-Louis Hospital, Paris, 75010 France; 3 INSERM UMR1342, Université Paris Cité, Paris, 75010 France; 4 Division of Plastic and Reconstructive Surgery, Cedars-Sinai Medical Center, Los Angeles, CA 90048 USA; 5 Department of Genetics, Institut Curie, Paris, France; 6 AP-HP, Hematology Department, European Georges Pompidou Hospital, Paris, 75015 France; 7 Inserm U1016, Paris City University, Paris, France; 8 Plateforme d’Imageries du Vivant PIV-ADI, Paris Cité University, Paris, France

**Keywords:** MSCs, Adipogenic differentiation, Wharton’s jelly, Bone marrow, Metabolic regulation

## Abstract

Mesenchymal stromal/stem cells (MSCs) derived from distinct tissue niches exhibit marked functional heterogeneity, yet the determinants governing their adipogenic competence remain incompletely understood. Wharton’s jelly MSCs (WJ-MSCs) are frequently considered poorly adipogenic compared with bone marrow MSCs (BM-MSCs), although the contribution of culture conditions to this phenotype has not been systematically investigated. Here, integrating a structured review of 41 studies with experimental modeling, we demonstrate that adipogenic commitment of WJ-MSCs is highly dependent on metabolic induction conditions and exposure duration. Structured literature review revealed substantial heterogeneity in adipogenic protocols across the literature, with no consensus framework for WJ-MSCs differentiation. Guided by these observations, we compared four adipogenic conditions differing in glucose availability and insulin supplementation and quantified differentiation using machine learning–assisted lipid imaging. Whereas BM-MSCs readily underwent adipogenesis under conventional induction conditions, WJ-MSCs displayed delayed and limited differentiation. However, prolonged induction combined with enhanced metabolic support revealed a delayed but significant adipogenic responsiveness for WJ-MSCs. High glucose and insulin synergistically promoted lipid accumulation in both MSCs sources, with the strongest effects observed under combined conditions. These findings identify metabolic context as a critical regulator of MSCs lineage plasticity and establish a standardized framework for evaluating adipogenic differentiation across tissue-derived MSCs populations.

**Graphical abstract:**

**Figure:**
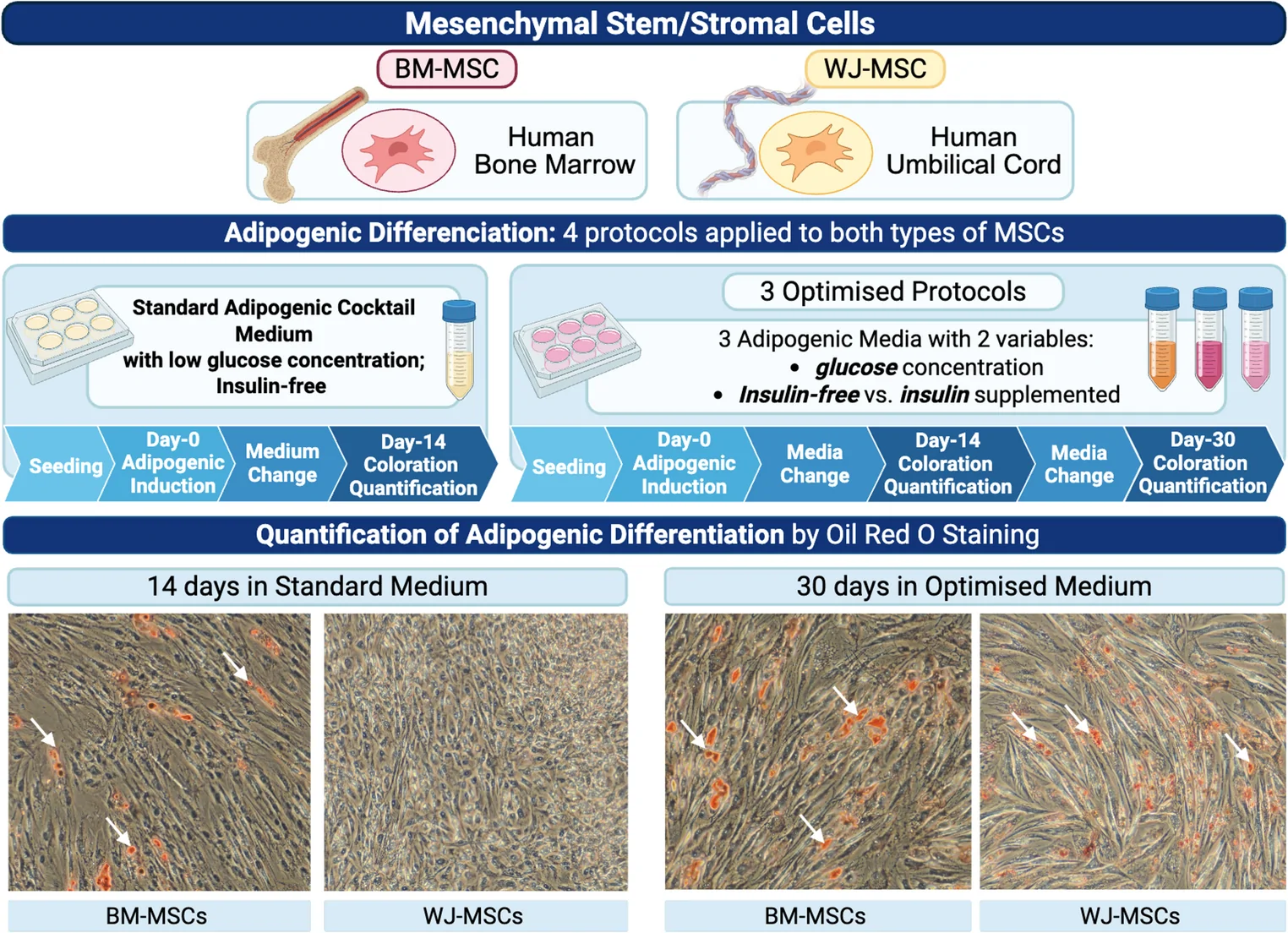
The Adipogenic Capacity of Wharton's Jelly MSCs Is Revealed by Optimized Culture Conditions. Created in BioRender. Smadja, D. (2026) https://BioRender.com/2o9112s

## Introduction

Mesenchymal stromal/stem cells (MSCs) have emerged as central players in regenerative medicine owing to their proliferative capacity, immunomodulatory properties, and multilineage differentiation potential. Originally identified in bone marrow, MSCs can now be isolated from a wide range of adult and perinatal tissues, including adipose tissue and the umbilical cord matrix [1, 2]. Despite sharing the minimal phenotypic criteria established by the International Society for Cell and Gene Therapy (ISCT) [3], MSC populations derived from distinct tissue niches exhibit profound functional heterogeneity, suggesting that tissue origin imprints lineage-specific differentiation programs and metabolic identities [4]. Among available MSC sources, Wharton’s jelly-derived MSCs (WJ-MSCs) have attracted considerable interest because of their high proliferative capacity, low immunogenicity, and accessibility without invasive procedures [5–8]. These properties position WJ-MSCs as particularly promising candidates for large-scale clinical applications in regenerative medicine. However, unlike bone marrow-derived MSCs (BM-MSCs) or adipose-derived stromal/stem cells (ADSCs), WJ-MSCs consistently display limited and highly variable adipogenic differentiation in vitro [9–11]. Across studies, adipogenic induction of WJ-MSCs is frequently characterized by sparse lipid droplet formation, delayed differentiation kinetics, and incomplete activation of canonical adipogenic transcriptional programs, even after prolonged induction periods extending up to 60 days [10, 12, 13]. Whether this phenotype reflects intrinsic lineage restriction or instead results from inadequate differentiation conditions remains unresolved.

Beyond its biological interest, adipogenic differentiation represents one of the three canonical mesenchymal differentiation pathways recommended by the International Society for Cell and Gene Therapy (ISCT) for the functional characterization of MSCs [3]. Consequently, robust assessment of adipogenic differentiation is not intended as a therapeutic endpoint itself but rather as a critical quality attribute for MSCs characterization and manufacturing. As WJ-MSCs are increasingly developed for advanced cell therapies because of their favorable proliferative and immunomodulatory properties [5–7], defining standardized adipogenic differentiation conditions is important to ensure reproducible quality control and to distinguish genuine biological differences from protocol-dependent variability. A major limitation in the field is the striking lack of standardization of adipogenic induction protocols across MSCs studies. Variability in glucose concentration, insulin supplementation, induction duration, and analytical readouts substantially complicates comparisons between tissue-derived MSCs populations and may obscure their true differentiation potential [14]. Emerging evidence further suggests that metabolic context critically regulates stem cell fate decisions [15, 16], raising the possibility that adipogenic competence of WJ-MSCs may depend on specific metabolic requirements distinct from those of adult MSCs.

Here, combining a structured review of 41 published studies with experimental modeling, we systematically investigated the adipogenic differentiation capacity of WJ-MSCs compared with BM-MSCs under defined metabolic conditions. Using optimized adipogenic media, machine learning–assisted lipid quantification, and transcriptional analyses, we identify glucose availability and insulin supplementation as major determinants of adipogenic commitment and demonstrate that prolonged metabolic induction unmasks an unexpected adipogenic competence in WJ-MSCs.

We hypothesized that the reduced adipogenic differentiation generally attributed to WJ-MSCs is not solely an intrinsic property of these cells but may instead result from non-optimized metabolic induction conditions. We therefore sought to determine whether systematic optimization of adipogenic culture conditions could reveal a previously underestimated adipogenic potential in WJ-MSCs while providing a standardized framework for evaluating adipogenesis across MSCs sources.

## Materials and Methods

### Methods Allocated to the Literature Review

A structured literature review was performed to compile a summary table of studies on the in vitro adipogenic differentiation of human MSCs (hMSCs). Tables include key details such as study title, publication year, MSCs tissue source, study design (cell seeding density, differentiation duration, medium change frequency), differentiation medium composition, analytical methods, and conclusions. The collected data led to the development of a proposed adipogenic cocktail and an analytical strategy for quantifying differentiation. The literature search was conducted through PubMed and Google Scholar, excluding studies published before 2000 and those not in English. Google Scholar was additionally searched to minimize publication bias and identify studies not indexed in PubMed. Keywords and Boolean operators were used to focus on articles related to adipogenic differentiation of hMSCs.

- (((mesenchymal stem cell) OR (mesenchymal stromal cell)) AND (adipogenic)) AND (human))
- ((Wharton jelly mesenchymal stromal cell) AND (adipogenic)) AND (human))
- (((Bone marrow mesenchymal stem cell) OR (bone marrow mesenchymal stromal cell)) AND (adipogenic)) AND (human))

Following the database search, duplicate records were removed before screening. Studies were eligible for inclusion only if they fulfilled the following criteria: (i) human mesenchymal stromal/stem cells were investigated; (ii) the adipogenic differentiation medium was described in sufficient detail; (iii) the adipogenic induction and maintenance protocol was reported; and (iv) the experimental design allowed comparison of differentiation conditions. Studies using non-human cells, lacking methodological details, published before 2000, or not written in English were excluded. A total of 41 studies fulfilled these criteria and were summarized in Tables 1, 2, 3 and 4. One publication was retained despite incomplete reporting of the differentiation medium because it provided a comprehensive description of an innovative analytical method directly relevant to the objectives of this review. This study is a narrative (non-systematic) review and was not conducted according to PRISMA guidelines. As such, no formal systematic search strategy, protocol registration, or risk-of-bias assessment was undertaken. The review aims to provide a qualitative overview of the current evidence rather than a comprehensive, systematic synthesis, and the findings should therefore be interpreted considering these methodological limitations.

**Table 1:** Adipogenic differentiation capacity of adipose-derived MSCs across published studies (8 studies)

Study of the Adipogenic Potential of ADSCs (8 studies)				
Reference	Study Design	Adipogenic Cocktail Composition	Analytical Methods	Results
Optimization of a culture medium for the differentiation of preadipocytes into adipocytes in a monolayer [27] France, 2009	40,000 cells/cm² T25 P3 Change every 3 days, during 21 days	• DMEM Glutamax/Ham’s F12 1:1 • 10% SVF • ATB : 20 µg/mL gentamycin + 100 UI/ml penicillin • 10 nM hydrocortisone, • 500 nM DEX • 0.5 mM IBMX • 0.15 UI/ml) insulin • 1 µM rosiglitazone	Adiponectin evaluation	Final optimized differentiation medium for adipocytes ADSCs: DMEM/HAMF12 10% FCS 2 nM triiodothyronine 10 nM hydrocortisone 0,5 mM IBMX 500 nM DEX 1 µM rosiglitazone 0,15 UI/ml insulin Antibiotics
Angiogenic Capacity of Human Adipose-Derived Stromal Cells During Adipogenic Differentiation: An In Vitro Study [49] Netherlands, 2009	2.0 10^4^cells/cm² in 6-well plates The medium was replaced every 2–3 days and 24 h prior to each measurement. Differentiation was evaluated on days 0, 3, 7, 14, and 22.	DMEM-HG 10% FBS 1 mM DEX 0.01 mg/mL insulin, 0.2mM INDO 0.5mM IBMX 1% penicillin/streptomycin 0.5% gentamycin 37 °C, 5% CO_2_	Adipogenic differentiation: Oil Red O (ORO) staining and quantification of % differentiated cells/total cell number using ImageJ software GAPDH activity measurement by spectrophotometry at 340 nm FABP4 expression analysis Angiogenic potential assessment: RT-PCR: VEGF, FGF-2, PGF, PDGFB, TGFB1, ANGPT1, ANGPT2 ELISA sandwich: VEGF, PLGF, ANG-1, ANG-2	Short-term adipogenic differentiation (7–14 days) of ADSCs enhances their angiogenic potential, evidenced by: Upregulation of pro-angiogenic genes (VEGF, PlGF, ANGPT-1, ANGPT-2) Increased VEGF protein secretion Improved viability of endothelial cells in vitro (via conditioned medium) This suggests that brief adipogenic priming of hASCs could promote graft vascularization.
Comparison between pediatric and adult adipose mesenchymal stromal cells [50] France, 2017	4000 cells/cm² 12-well plate Change media every 3 days For 14 days	• Induction for 3 days: • Expansion medium AS • 1 µmol/L DEX • 5 µg/mL insulin • 1 µmol/L rosiglitazone • 450 µmol/L IBMX • 60 µmol/L INDO Maintaining during 11 days, use the same medium without IBMX	ORO staining: Cellular triglyceride (TG) content was measured using a commercial test (Triglycerides Enzymatic PAP 150, Biomérieux). Protein content was determined using the DC protein assay kit (BioRad). Differentiation quantification was evaluated by calculating the ratio between TG and total protein content.	Similar adipogenic differentiation capacity between pediatric and adult ADSCs
Energy metabolic capacities of human adipose-derived mesenchymal stromal cells in vitro and their adaptations in osteogenic and adipogenic differentiation [51] Germany, 2018	In 4-well Plate 20,000 cells/cm² In 6-well plate and 96-well plate For 14 days	*Proliferation Medium*: 1 µM DEX • 500 µM IBMX • 200 µM INDO • 10 µM insulin • 1% penicillin/streptomycin • 10% FBS	Bodipy staining, measured in UFR (relative fluorescence). Quantification of cell number by OD measurement after crystal violet staining.	Adipogenic differentiation leads to a decrease in carbohydrate metabolism capacity.
Influence of glucose and insulin in human adipogenic differentiation models with adipose-derived stem cells [16] Germany, 2019	6-well plates For 28 days	• Standard differentiation medium • 1 µM DEX • 0,5 mM IBMX • 0,1 mM INDO • 1,7 µM hr insulin	ORO and hematoxylin staining Quantification using a Java ImageJ-based image processing program and spectrophotometry at 518 nm Live/dead fluorescent staining (EthD-1/Calcein AM) RTQ-PCR of LPL, PPAR-γ	• Glucose and insulin have no effect on viability. • Glucose increases lipid levels, but not significantly. • Insulin significantly reduces lipid accumulation.
Autofluorescence-Raman Mapping Integration analysis for ultra-fast label-free monitoring of adipogenic differentiation of stem cells [52] South Korea, 2021	5 × 10³ cells Seeded on quartz coated with fibronectin Change medium every 2 days For 2 weeks	*Commercial Kit*: StemPro adipogenesis differentiation kit medium (Gibco Life Technologies, Seoul, South Korea)	ORO staining Fluorescence staining with Bodipy Combination of micro-Raman spectroscopy and autofluorescence imaging technique ‘AF-Raman mapping integration’ (ARMI) qPCR for CEBP-α, PPAR-γ, LPL, FABP4 FACS for CD90, CD105, CD73, and CD34.	AF-guided Raman mapping allows for the identification of lipid droplets among all cellular components. ARMI analysis can accurately determine the differentiation levels of stem cells
A Comparative Study on the Adipogenic Differentiation of Mesenchymal Stem/Stromal Cells in 2D and 3D Culture [53] Germany, 2022	Change media every 2 or 3 days	• Complete culture media • 1 µM DEX • 500 µM d’IBMX • 200 µM INDO • 10 µM insulin		
Optimizing the Adipogenic Induction Protocol Using Rosiglitazone Improves the Physiological Parameters and Differentiation Capacity of Adipose Tissue-Derived Mesenchymal Stem Cells for Horses, Sheep, Dogs, Murines, and Humans [54] Germany, 2023	1 × 10^4^ cells/cm² 24-well plate For 7 to 14 days Change media every 3 or 4 days	• DMEM-HG + 5% FBS • ITS (10 µg/mL insulin, 0,55 µg/mL transferrin and 6,8 ng sodium selenite) • 1 µM DEX • 0,5 mM IBMX • 0,2 mM INDO DMEM-HG + 5% FBS • ITS • 1 µM DEX Various concentrations of rosiglitazone 1 µM (Rosi 1), 5 µM (Rosi 5), and 10 µM (Rosi 10).	ORO staining, semi-quantification by spectrophotometry at 492 nm. Morphometric analysis of adipocyte size. SRB and MTT assays for cell quantification at 565 nm and 570 nm. RTQ-PCR	5 µM rosiglitazone is a potent PPAR-γ2 agonist to safely stimulate adipogenesis in ADSCs. The widely used IBMX/INDO conditions increase the risk of apoptosis in the stem cell pool, thereby altering their differentiation potential

**Table 2:** Adipogenic differentiation efficiency and induction protocols in bone marrow–derived mesenchymal stromal cells (10 studies)

Studies on Adipogenic Differentiation from BM-MSCs (10 Studies)				
Reference	Study Design	Adipogenic Cocktail Composition	Analytical Methods	Results
Expansion of Human Adult Stem Cells from Bone Marrow Stroma: Conditions that Maximize the Yields of Early Progenitors and Evaluate Their Quality [55] USA, 2002	5,000 cells/cm² in six-well culture plates 21 days	• Complete medium • 0,5 µM DEX • 0,5 mM IBMX • 50 µM INDO	ORO Staining Absorbance measurement at 510 nm	The highest number of adipocytes was generated from cultures plated at lower densities for shorter durations.
Characterization of adipocyte differentiation from human mesenchymal stem cells in bone marrow [35] China, 2010	21 days with 3 cycles	1 induction cycle: For 3 days • IBMX • DEX • Insulin • For 1 day: insulin•	Oil Red O (ORO) Staining Western Blot analysis of FABP4 and GLUT4 expression qPCR quantification of PPAR-γ, C/EBPβ, and C/EBPα	Successful adipogenic differentiation of BM-MSCs was achieved
Human Mesenchymal Stem Cells as an in Vitro Model for Human Adipogenesis [23] USA, 2012	2 à 5 × 10^3^/cm² 21 days	3 cycles induction/maintain - *Induction medium* • DMEM-HG (4,5 g/L glucose) • 10% FBS • 1 µM DEX • 0,2 mM INDO • 1,7 µM insulin • 0,5 mM IBMX • ATB: 0,05 U/mL de penicillin and 0,05 µg/mL streptomycin - *Maintaining medium* • DMEM-HG (4,5 g/L glucose) • 10% FBS • 1,7 µM human recombinant insulin • ATB: 0,05 U/mL de penicillin and 0,05 µg/mL streptomycin)•	ORO staining, absorbance measurement at 500 nm RT-qPCR Quantification: Adipogenic markers: PPAR-γ, C/EBPβ Metabolic regulators: Leptin, GLUT4, Adipsin	Optimal Differentiation Protocol for BM-MSCs: The most effective medium utilizes a 3 + 3 protocol with: • 5% FBS • 170 nM insulin • 4.5 g/L D-glucose • Thiazolidinedione treatment (Note: “3 + 3” likely refers to a 3-day induction + 3-day maintenance cycle)
Assay validation for the assessment of adipogenesis of multipotential stromal cells - a direct comparison of four different methods [56] UK, 2013	4 × 10^4^ cells/cm^2^ 24-well plates or 48-well plates Change media to 3 days, for 21 days	• DMEM complete • 10% FBS • 10% horse calf • 50 pmol/L hydrocortisone, • 0,5 mmol/L IBMX • 0,06 mmol/L INDO, • 2 mmol/L de L-glutamine • Antibiotics•	ORO staining, absorbance measurement at 510 nm qPCR and flux cytometer: FABP4 Fluorescence assay	
Novel Function of Serine Protease HTRA1 in Inhibiting Adipogenic Differentiation of Human Mesenchymal Stem Cells via MAP Kinase-Mediated MMP Upregulation [22] Switzerland, 2016	10 000 cells/cm² 14 to 24 days	*Induction Medium* • DMEM-HG • 1 µM DEX • 10 µg/mL insulin • 0,1 mM INDO • 0,5 mM IBMX • *Maintaining medium*: Without IBMX, reconstructed every 72 h•	ORO staining, absorbance measurement at 510 nm RTQ-PCR	
Protocols for in vitro Differentiation of Human Mesenchymal Stem Cells into Osteogenic, Chondrogenic and Adipogenic Lineages [57] Italia, 2016	2 × 10^4^ cells/cm². In 6-well plate. Change of medium every 3 to 4 days for 3 weeks	DMEM HG + 10% FBS. 1% ATB 1 mM DEX 0.5 mM IBMX 50 µM INDO	ORO Staining	
Mesenchymal Stromal Cell Irradiation Interferes with the Adipogenic/Osteogenic Differentiation Balance and Improves Their Hematopoietic-Supporting Ability [58] Espagna, 2018	22.5 × 10^3^ Change media every 2 weeks	*Commercial Kit*: • human MSCs Adipogenic Maintenance SingleQuots (Lonza)	ORO staining	Low-dose irradiation decreases the adipogenic differentiation of human MSCs
HSPB7 oppositely regulates human mesenchymal stromal cell-derived osteogenesis and adipogenesis [59] Netherlands, 2023	Change media every 3 or 4 days	• 0,1 µM DEXA • 60 µM INDO • 0,5 mM IBMX	ORO, absorbance at 490 nm. Cell counting and normalization (calculation of individual cell absorbance). Adipogenic marker expression evaluated by RTQ-PCR. Adipogenic genes evaluated by Western blot	The silencing of HSPB7 promotes adipogenesis.
Abnormal adipogenic signaling in the bone marrow mesenchymal stem cells contributes to supportive microenvironment for leukemia development [60] Israel, 2023	8 days	• Basal medium: MEM-α with commercial adipogenic supplementation (R&D Systems, cat. #CCM011, Minneapolis, MN, USA)	ELISA assay for FABP4 from plasma Flow cytometry: primary antibody labeling for FABP4.	
Bone Marrow Adipocytes Contribute to Tumor Microenvironment-Driven Chemoresistance via Sequestration of Doxorubicin [61] USA, 2023	96-well plate 80% cell confluence to induce with AM1 for 9 days Followed by a maintenance phase with AM2 for 9 days Differentiation for 18 days Medium change every 3 days	• *Induction Medium (AM1)* • DMEM-HG + 10% (v/v) FBS • 1% (v/v) de PS, • 1 µM DEX • 500 µM IBMX • 10 µM rosiglitazone • 10 µg/mL insulin *Maintaining medium* DMEM-HG + 10% (v/v) FBS, 1% (v/v) PS 10 µg/mL insulin•	BODIPY staining, phalloidin-TRITC, and DAPI staining Measurement of lipid droplet diameter change	The addition of rosiglitazone significantly accelerates adipogenesis and the formation of mature lipid droplets compared to conventional adipogenic differentiation medium in human BM-MSCs.

**Table 3:** Adipogenic differentiation protocols and outcomes in Wharton’s jelly–derived mesenchymal stromal cells (12 studies)

Studies on Adipogenic Differentiation from WJ-MSCs (12 Studies)				
Reference	Study Design	Adipogenic Cocktail Composition	Analytical Methods	Results
Critical Parameters for the Isolation of Mesenchymal Stem Cells from Umbilical Cord Blood [13] Germany, 2004	Confluence in eight-chamber slides 3 cycles + 7 days of incubation in maintenance medium	According to the protocol of *Pittenger et al.* [21]. - 3 cycles of adipogenic induction/maintenance. *Induction medium* • 10% FBS • 1 mM DEX • 0,5 mM IBMX • 100 mM INDO • 10 µg/mL hr insulin - *Maintenance medium* • Containing only 10 µg/mL hr insulin and 10% FBS	ORO Staining Nuclei stained with hematoxylin solution	Adipocytes could only be detected by ORO staining after intense induction for at least 5 weeks.
Characterization of mesenchymal stromal cells derived from full-term umbilical cord blood [24] UK, 2008	2 weeks	DMEM-HG • 10% FBS • 0,5µM hydrocortisone • 0,5mM IBMX • 0,06 mM INDO • Antibiotics	ORO Staining	None of the tested cell lineages differentiated into adipocytes.
Are serum-free and xeno-free culture conditions ideal for large scale clinical grade expansion of Wharton’s jelly derived mesenchymal stem cells? A comparative study [39] India, 2014	18 days	• DMEM KO • 10% FBS • 200 mM INDO • 0,5 mM IBMX • 10 mg/mL insulin • 1 mM DEX•	ORO staining, absorbance measurement at 540 nm	Successful adipogenic differentiation
The effect of fibroblast growth factor on distinct differentiation potential of cord blood–derived unrestricted somatic stem cells and Wharton’s jelly–derived mesenchymal stem/stromal cells [29] South Korea, 2015	3 × 10^4^ cells/cm² 12-well plates 3 weeks	Commercial adipogenic induction medium: Gibco. With and without supplementation of 10 ng/mL bFGF.	ORO staining RTQ-PCR - SYBR Green: FABP4, LPL, PPAR-γ2	Very little ORO staining. Low expression level of FABP4 and LPL in WJ-MSCs, PPAR-γ highly expressed. Supplementation with bFGF had no positive effect on the tri-lineage differentiation potential of WJ-MSCs in this study
Multi-Lineage Differentiation of Human Umbilical Cord Wharton’s Jelly Mesenchymal Stromal Cells Mediates Changes in the Expression Profile of Stemness Markers [62] Kuwait, 2015	24 well plate 2 × 10^4^ cells/well Culture medium was replaced every 3 days for 21 days	DMEM-low glucose 10% FBS ATB 1µM DEX 500 µM IBMX 5 µg/ml insulin 200 µg/ml ascorpate-2-phosphate	ORO staining Cells were harvested at days 7, 14 and 21 for RNA extraction and RTQ-PCR analysis	Adipocyte formation, positive Oil Red O staining, intracellular lipid droplets qRT-PCR: time-dependent upregulation, adipogenic markers, PPAR-γ, adiponectin, vs. non-differentiated controls
IGFBP2 enhances adipogenic differentiation potentials of mesenchymal stem cells from Wharton’s jelly of the umbilical cord via JNK and Akt signaling pathways. *PloS One* [30] China, 2017	3 weeks	Commercial adipogenesis differentiation kit StemPro (Invitrogen, Carlsbad, CA, USA)	ORO Staining RTQ-PCR: IGFBP2, PPAR-γ, LPL, CEBPα,	Overexpression of IGFBP-2 enhances adipogenic differentiation in WJ-MSCs.
Isolation and Characterization of Mesenchymal Stromal Cells from Human Umbilical Cord and Fetal Placenta [63] USA, 2017	100 000 cells / well	• 1 µM DEX • 0,5 µM IBMX • 200 µM INDO • 10 µM insulin • Change media every 3 days	ORO staining	The results show that the quality and quantity of the isolated MSCs differ between the four sources of the cord/placenta sample.
Evaluation of reference genes for quantitative real-time PCR in Wharton’s Jelly-derived mesenchymal stem cells after lentiviral transduction and differentiation [64] Pologne, 2019	21 days	• *Commercial Kit*: StemMACS AdipoDiff (Miltenyi Biotec ; 130-091-677)	ORO Staining	A few lipid vacuoles are detected using ORO staining
Efficient differentiation of vascular smooth muscle cells from Wharton’s Jelly mesenchymal stromal cells using human platelet lysate: A potential cell source for small blood vessel engineering [65] Greece, 2020	1 × 10^5^ cells 6-well plate	*Commercial Kit*: StemPro Adipogenesis (ThermoFischer Scientific, Massachusetts, États-Unis)	ORO staining	WJ-MSCs successfully produced lipid vacuoles, visible with ORO staining.
Metformin potentiates immunosuppressant activity and adipogenic differentiation of human umbilical cord-mesenchymal stem cells [66] Italia, 2023	21 days, Change media every 3 days	• *Commercial Kit*: • StemPro™ Adipogenesis Differentiation Kit (Gibco, ThermoFisher Scientific), the medium is replaced with metformin treatment every 3 days.	ORO staining, quantification by ImageJ: (particle size from 0 to infinity), each image is measured after thresholding. The data are expressed as integrated density (IntDen) as the sum of the pixel values. QPCR	The commitment of UC-MSCs to adipogenic differentiation was potentiated by metformin (3 and 5 mM). Metformin induces the formation of lipid vacuoles during the osteogenic differentiation of UC-MSCs. 3 mM metformin increases PPAR expression and reduces FABP4 expression.
Identification of critical process parameters for expansion of clinical grade human Wharton’s jelly-derived mesenchymal stromal cells in stirred-tank bioreactors [22] Espana	1 × 10^4^ cells/cm² 24-well plate for 35 days	• *Commercial Kit*: StemPro (Gibco) • Antibiotics: 100 U/mL of penicillin and 100 µg/mL of streptomycin	ORO Staining	Commitment of WJ-MSCs to the adipogenic lineage confirmed
A GMP-compliant manufacturing method for Wharton’s jelly-derived mesenchymal stromal cells [41] China, 2024	6-well plate exchanged every 3 days 4-weeks	• *Commercial medium*: • OriCell™ Adipogenic Differentiation Medium for Human Umbilical Cord Mesenchymal Stem Cells•	ORO Staining	The P1 and P5 WJ-MSCs demonstrated multilineage differentiation potential, successfully differentiating into osteogenic, adipogenic, and chondrogenic lineages.

**Table 4:** Tissue origin defines adipogenic differentiation capacity across mesenchymal stromal cell populations (17 studies)

Comparison of the Adipogenic Potential of MSCs According to Their Tissue of Origin (17 Studies)					
Reference	Type of MSCs	Study Design	Adipogenic Cocktail Composition	Analytical Methods	Results
Disparate Mesenchyme-Lineage Tendencies in Mesenchymal Stem Cells from Human Bone Marrow and Umbilical Cord Blood [36] Taiwan, 2006	BM-MSCs UCB-MSCs	7 × 10^3^ cells/cm^2^ In six-well plates 3 à 5 weeks	• α-MEM • 10% SVF • 1 µM DEX • 0,5 mM IBMX • 100 µM INDO • 10 µg/mL insulin	ORO Staining Absorbance measurement at 550 nm RTQ-PCR	The induction of adipogenesis in cord blood MSCs resulted in < 5% ORO-positive cells, with smaller lipid droplets compared to BM-MSCs.
Comparative Analysis of Mesenchymal Stem Cells from Bone Marrow, Umbilical Cord Blood, or Adipose Tissue [11] Germany, 2006	BM-MSCs UC-MSCs ADSCs	2,1 × 10^4^ cells/cm^2^ In eight-chamber slides 3 cycles + 7 days of incubation in maintenance medium	Culture in MSC-GM or DMEM-LG + 10%MSCGS According to the protocol of Pittenger et al. [21]. - 3 cycles of adipogenic induction/maintenance. - *Induction medium* • 10% SVF • 1 mM DEX • 0,5 mM IBMX • 100 mM INDO • 10 µg/mL insulin hr - *Maintenance medium* • Containing only 10 µg/mL hr insulin and 10% FBS	ORO Staining Nuclei stained with hematoxylin solution	
Biology of Stem Cells in Human Umbilical Cord Stroma: In Situ and In Vitro Surveys [67] Turkey, 2006	UC-MSCs WJ-MSCs BM-MSCs	P3 to P7 In six-well plates 6 weeks	• DMEM-LG • 10% SVF • 1 µM DEX • 500 µM IBMX • 60 µM INDO 5 µg/ml insulin	ORO Staining Nuclei stained with hematoxylin solution Absorbance measurement at 540 nm RTQ-PCR PAI-1	Adipogenic differentiation of WJ-MSCs after 40 days, without acquisition of a mature adipocyte phenotype
Replicative senescence of human bone marrow and umbilical cord derived mesenchymal stem cells and their differentiation to adipocytes and osteoblasts [12] China, 2010	BM-MSCs UC-MSCs	1 × 10^5^ cells/well in 6-well plates 21 days	• DMEM • 10% FBS • 1 mM DEX • 0,5 mM IBMX • 200 µM INDO • 10 mM insulin	ORO staining, Absorbance measurement at 490 nm qPCR quantification: CEBP-α, PPAR-γ, ALPL	Lipid vesicles were significantly smaller in WJ-MSCs compared to BM-MSCs PPAR-γ expression decreased while ALPL increased CEBP-α did not regulate differentiation in WJ-MSCs
Adipogenic Differentiation of Human Mesenchymal Stem Cells [68] Denmark, 2011	BM-MSCs ADSCs	30 000 cells/well 48-well plates 3 weeks	• 0.45 mM IBMX • 0.2 mM INDO • 0.1mM DEX • 1 µM Rosiglitazone • 1 µg/mL insulin	ORO staining Fluorescence Staining: Hoechst 33,342 (nuclear staining) BODIPY 493/503 (lipid droplet visualization) Gene Expression Analysis: RT-PCR for adipogenic markers: PPAR-γ, aP2. Reference genes: (specify if needed, e.g., GAPDH, β-actin)	Lipid droplet formation and adipogenic gene transcription were detectable as early as the second week of adipogenic treatment.
Distinct Differentiation Potential of ‘‘MSC’’ Derived from Cord Blood and Umbilical Cord: Are Cord-Derived Cells True Mesenchymal Stromal Cells? [69] Germany, 2012	BM-MSCs UC-MSCs CB-MSCs Fibroblast	plated at 8.3 × 10^3^ cells/cm² 6-well plates changed media twice a week for 21 days	Alternating induction and cultivation medium: DMEM high glucose (Lonza) 10% FCS PSG 10^− 6^ M DEX 0.2mM INDO 0.1 mg/mL insulin 1mM IBMX The latter was made up of DMEM high glucose, 10% FCS, and 0.01 mg/mL insulin.	Stained with Oil Red O Images were analyzed using the ImageJ Java-based image processing software for windows. RT-PCR and real-time-PCR Western blot Flow cytometry	Cells isolated from human umbilical cord lack adipogenic, osteogenic, and chondrogenic differentiation potential and therefore do not meet the definition of mesenchymal stromal cells. Differentiation can be efficiently assessed by combining gene expression analysis with staining methods. HOX gene expression and CD56/CD146 markers distinguish UC cells from those derived from other tissue sources.
Isolation and Characterization of Human Mesenchymal Stem Cells Derived from Human Umbilical Cord Wharton’s Jelly and Amniotic Membrane [70] IRAN, 2013	WJ-MSCs AM-MSCs	NA	Commercial Medium (Bonyakhteh, Iran)	ORO staining Pictures x100 RT-PCR	
Comparison of different methods for the isolation of mesenchymal stem cells from umbilical cord matrix: proliferation and multilineage differentiation as compared to mesenchymal stem cells from umbilical cord blood and bone marrow [43] China, 2013	WJ-MSCs UCB-MSCs BM-MSCs	2.1 × 10^3^ cellscm² 6-well plates Three cycles of induction/maintenance, followed by 7 days of incubation in adipogenic differentiation medium (changed every 3 days) → 28 days	• DMEM-LG • 10% de FBS • Use medium for 3 days • 1 mM DEX • 0,5 mM IBMX • 10 µg/mL insulin • 100 mM INDO • 10% de FBS • Use maintaining medium for 4 days • 10 µg/mL insulin + 10% de FBS	ORO staining + counterstaining with Mayer’s hematoxylin → Evaluation of differentiation by examination/analysis of 10 random high-power fields (400×).	WJ-MSCs and BM-MSCs exhibited adipogenic differentiation. BM-MSCs showed a higher adipogenic differentiation rate than WJ-MSCs.
Protein synthesis and secretion in human mesenchymal cells derived from bone marrow, adipose tissue and Wharton’s jelly [38] Brazil, 2014	BM-MSCs ADSCs WJ-MSCs	In 24-well plate 13157cells/cm² 21 days	LG-DMEM 10% FBS 1 µM DEX 0,5 mM IBMX 10 µM human insulin 0,2 mM INDO ATB penicillin/streptomycin à 100 U/mL et 100 µg/mL Change media every 2 days	ORO staining Dosage of angiogenic factors in the supernatants of each cell type	ADSCs and BM-MSCs exhibited a higher adipogenic potential compared to WJ-MSCs, despite having a higher secretion of Thrombospondin-2 and Endostatin (with no VEGF secretion).
Gene expression and protein secretion during human mesenchymal cell differentiation into adipogenic cells [40] Brazil, 2014	WJ-MSCs ADSCs BM-MSCs	NA	• CSMs cultured in DMEM supplemented with FBS and the recommended compounds for adipogenic induction. Differentiation controls maintained in standard medium were run in parallel for all conditions, and they were all negative, showing no visible lipid accumulation.	Expression of adipogenic genes (PPARG, ADIPOQ, and CEBP-α) and the reference gene RPL13A by quantitative real-time PCR. Secretion of cytokines, extracellular matrix (ECM) components, and growth factors analyzed by Luminex and ELISA.	MSCs from different tissues exhibit different behavior when induced to differentiate into adipocyte-like cells.
Multi-Lineage Differentiation of Human Umbilical Cord Wharton’s Jelly Mesenchymal Stromal Cells Mediates Changes in the Expression Profile of Stemness Markers [62] Kuwait 2015	WJ-MSC	24 well plate 2 × 10^4^ cells/well Culture medium was replaced every 3 days for 21 days	DMEM-low glucose 10% FBS ATB 1µM DEX 500 µM IBMX 5 µg/ml insulin 200 µg/ml ascorpate-2-phosphate	ORO staining Cells were harvested at days 7, 14 and 21 for RNA extraction and qRT-PCR analysis	Adipocyte formation, positive Oil Red O staining, intracellular lipid droplets qRT-PCR: time-dependent upregulation, adipogenic markers, PPAR-γ, adiponectin, vs. non-differentiated controls
Improved adipogenic in vitro differentiation: comparison of different adipogenic cell culture media on human fat and bone stroma cells for fat tissue engineering [71] Germany, 2015	BM-MSCs ADSCs	2500 cells/cm² 6-well plate 21 days	- - *Base medium* • DMEM-LG • 500 nM DEX • 500 µM IBMX • 50 µM INDO • 0.1UI/mL(3.47 µg/mL) insulin - *Specific differentiation medium*: *Basal supplementation*: • DMEM/Ham’s F-12 • 10% FBS • ATB: 100 IU/ml penicillin, 100 µg/ml streptomycin • 2 mM L-glutamine • 1 mM ascorbate-2-phosphate - *Specific supplementation*: • 2 nM triiodothyronine, • 10 nM hydrocortisone, • 500 µM IBMX, • 500 µM DEX, • 1 µM rosiglitazone 0.15 UI/mL insulin (5.205 µg/mL)	ORO staining, counting with a hemocytometer. RT-qPCR for the genes: C/EBPα, LPL, GLUT4, PPAR-γ2, and FABP4.	The authors recommend using a specific differentiation medium for adipogenic differentiation from other cell types.
IGFBP2 enhances adipogenic differentiation potentials of mesenchymal stem cells from Wharton’s jelly of the umbilical cord via JNK and Akt signaling pathways. *PloS One* [30] China, 2017	WJ-MSCs	3 weeks	Commercial adipogenesis differentiation kit StemPro (Invitrogen, Carlsbad, CA, USA)	ORO Staining RTQ-PCR : IGFBP2, PPAR-γ, LPL, C/EBPα	Overexpression of IGFBP2 enhances adipogenic differentiation in WJ-MSCs.
Comparison of in vitro-cultivation of human mesenchymal stroma/stem cells derived from bone marrow and umbilical cord [4] Germany, 2017	BM-MSCs UC-MSCs	15 000–30 000 cells/cm^2^ 14 days	• DMEM-LG (1 g/l) • 10% FBS • 20 mm acid hydroxyethyl piperazinethanesulfonique (HEPES), • 1 µM DEX • 500 µM IBMX • 60 µM INDO - 10 µg/mL insulin-	ORO Staining Nile Red O or Bodipy fluorescence staining Analysis of gene products: aP2, FABP4, LPL, PPAR-γ, SREBP1	
Visualization and Quantification of Mesenchymal Cell Adipogenic Differentiation Potential with a Lineage Specific Marker [72] Australia, 2018	BM-MSCs ADSCs	5.10^3^ cells/well Change media every 2 or 3 days	• ASC complete • 1 µM DEX • 200 µM INDO • 10 µM insulin	ORO staining Immunofluorescent de FABP4	
Comparative evaluation of mesenchymal stromal cells from umbilical cord and amniotic membrane in xeno-free conditions [73] China, 2018	UC-MSCs (umbilical cord) AM-MSCs (amniotic membrane)	Passage 5	• *Commercial Kit*: • StemPro Adipogenesis (Gibco, Grand Island, NY, USA)	ORO Staining RTQ-PCR PPAR-γ and LPL	AM-MSCs in platelet lysate showed similar adipogenic differentiation potential to UC-MSCs
Enhancing adipogenesis in Wharton’s jelly multipotent mesenchymal stromal cells through lipidomic insights and fatty acid supplementation [15] Czech Republic, 2025	WJ-MSCs AT-MSCs	Cells from passages 3–5 Cells density of 5 × 10^3^ cells/cm² 21 days The medium was replaced every 3 days.	*Adipogenic media*: α-MEM 10% FBS penicillin/streptomycin GlutaMAX 0.5 mM IBMX 0.1 µM DEX 0.1 mM INDO 10 µg/ml insulin *Optimized media*: The induction medium was additionally supplemented by 100 µM Oleic Acid or 100 µM Linoleic acid or a mixture 50 µM each.	Nile red staining and fluorescence microscopy Lipidomic profile of cells was explored by the liquid chromatography-mass spectrometry Quantitative real-time PCR (C/EBPα; PPARG; FABP4; LPL)	WJ-MSCs and AT-MSCs exhibit distinct adipogenic differentiation efficiencies, due to differences in their initial triglyceride profiles and responses to standard inducers. Supplementation with monounsaturated oleic acid enhances adipogenic differentiation of WJ-MSCs, improving their potential for soft tissue engineering applications.

### Human MSCs Isolation and Culture

Bone marrow and umbilical cord were both obtained and isolated from healthy donors through the Biological Resource Center of the Cell therapy Unit at Saint-Louis Hospital, Paris. Healthy donors provided their consent for the use of their biological material in research. The isolation and expansion of BM-MSCs and WJ-MSCs were performed as previously described [5, 17]. Cryopreservation is carried out using medium consisting of 90% fetal bovine serum (FBS) with 10% DMSO. Cryovials containing between 5 × 10^5^ and 1 × 10^6^ cells were frozen using a protocol that allowed for the gradual decreases of the temperature and were stored in liquid nitrogen until use. After thawing, BM-MSCs are seeded into 25 cm² culture flasks in complete Minimum Essential Medium Alpha (1X) supplemented with GlutaMAX™-I, 10% FBS, penicillin/streptomycin, and 10 ng/mL fibroblast growth factor (bFGF), and maintained under standard cell culture conditions (humidified atmosphere with 5% CO₂ at 37 °C). The medium is replaced every two days until confluence is reached, after which the cells are dissociated using trypsin and seeded into 6-well plates at a density of 1 × 10⁵ cells per well. For WJ-MSCs, thawing is performed in NutriStem^®^ XF medium (Sartorius) supplemented with 10% FBS. WJ-MSCs exhibit a high proliferative capacity, and confluence may be reached earlier than for BM-MSCs. Depending on the number of passages the cells have undergone, the cells are either passaged further to increase expansion or transferred into 6-well plates at a density of 1 × 10⁵ cells per well. The medium is changed every two days until confluence reaches approximately 90%.

### Adipogenic Differentiation of BM-MSCs and WJ-MSCs

When cell confluence reached > 90%, the proliferation medium (αMEM for BM-MSCs and NutriStem^®^ XF for WJ-MSCs) was replaced with adipogenic differentiation media. The standard adipogenic condition used in this study, referred to as LGI- (Low Glucose, Insulin-free), served as the reference condition. This medium consisted of DMEM Low Glucose (1.0 g/L glucose; Gibco, 11880-028) supplemented with 10% FBS (PAN-Biotech, P30-3306) and 1% penicillin/streptomycin (100 U/mL penicillin and 100 µg/mL streptomycin; Invitrogen). This basal medium was further enriched with a classical adipogenic induction cocktail composed of dexamethasone (10⁻⁶ M), indomethacin (60 µM), and isobutylmethylxanthine (IBMX, 0.5 mM). To investigate the impact of glucose and insulin on adipogenic differentiation, three additional media were formulated by varying glucose concentration (1.0 g/L vs. 4.5 g/L) and insulin supplementation (absence vs. 10 µg/mL). High-glucose conditions were achieved using DMEM High Glucose (4.5 g/L glucose; Gibco, 61965-026), while maintaining identical supplementation with FBS, antibiotics, and the adipogenic cocktail. Recombinant human insulin (Sigma, I2643) was prepared as a 2 mg/mL stock solution in hydrochloric acid (666 mmol/L) and added to the relevant media at a final concentration of 10 µg/mL.

This experimental design resulted in four distinct adipogenic conditions:

- **LGI- (Low Glucose**,** Insulin-free)**: DMEM Low Glucose + adipogenic cocktail, without insulin.
- **LGI+ (Low Glucose**,** Insulin)**: DMEM Low Glucose + adipogenic cocktail + insulin (10 µg/mL).
- **HGI- (High Glucose**,** Insulin-free)**: DMEM High Glucose + adipogenic cocktail, without insulin.
- **HGI+ (High Glucose**,** Insulin)**: DMEM High Glucose + adipogenic cocktail + insulin (10 µg/mL).

All differentiation media were renewed every 3 days throughout the culture period over 14 or 30 days, with a fifth condition included as a proliferation control, where cells seeded under identical conditions are maintained with αMEM. Before staining, cells are imaged to document their shape and lipid droplet formation using an optical microscope at various magnifications and phase contrasts with a Nikon N16184 camera. Illustrative images, edited in PowerPoint without altering information, are non-analytical.

## Oil Red O Staining of Triglyceride Droplets

At days 14 and 30 of adipogenic differentiation, cell cultures are washed with calcium- and magnesium-free PBS-/- (Eurobioscientific, CS1PBS01-01) at 37 °C and fixed for 30 min in 4% formaldehyde diluted in PBS-/- at room temperature. Fixed wells are rinsed with PBS-/- and incubated for 30 min with 1 mL of 0.3% Oil Red O (ORO) solution. Next, the ORO solution is removed, and the wells are gently washed with Milli-Q water, until the rinse solution is clear and free of aggregates. The plates are then dried for at least 2 h prior to imaging under a chemical fume hood to allow evaporation of residual isopropanol. ORO solution is prepared freshly in Milli-Q water from a 0.5% isopropanol stock (Sigma, 01391), allowed to settle for at least 30 min, and then filtered (0.22 μm, ClearLine, 037044) to minimize aggregates.

### Representative Photo of Neutral Lipid Staining

After staining, the images were taken to illustrate adipogenic differentiation results and provide data for quantifying the stained pixels in the images with the FIBER-ML software [18]. Images are captured with an optical microscope (Nikon N16184) at different magnifications and phase contrasts. Contrast and brightness adjustments in PowerPoint enhance clarity without altering information, highlighting clear differentiation.

### Photo for FIBER-ML Quantification

To ensure accurate representation of differentiation within a well, a plastic grid was placed under the plate during imaging to avoid capturing the same area more than once. The grid, measuring 2 mm × 4 mm, spans the 3.5 cm diameter of a 6-well plate. This method ensures consistent microscope objective movement across the well, reducing operator bias and enabling efficient imaging of distinct fields. 10 images per well are captured with a ×10 objective lens using “PhL” phase contrast, producing a gray background, gray undifferentiated cells, and bright red differentiated cells. Photos are transferred the same day to an external hard drive and systematically renamed to include details like cell type, culture time, medium, replicate number, and photo number.

## Analysis Using FIBER-ML

FIBER-ML is a semi-automated, open-source software based on a machine learning approach, designed to automatically quantify the proportion of differentiated areas following a brief user-guided training phase. FIBER-ML version 1.0 (https://gitlab.com/balvayda/fiber-ml, last accessed January 11, 2022), available free of charge under the GNU license, was developed using Matlab (MathWorks, Natick, MA) to enable simple and efficient segmentation and classification of images, as well as the quantification of pixels corresponding to newly formed lipids stained in red. As previously described [18, 19], the core of the software consists of a training interface coupled with an automated segmentation module. Training is performed by manually selecting pixels within the images corresponding to different types of tissues. This numerical sample is provided to the classification module that relies on a low-complexity classifier based on discriminant analysis to generalize the user decisions to every pixel in the images. FIBER-ML also includes a study management module, allowing for the tracking and reuse of data generated throughout the process, as well as a quality control interface. The software can be used either as a standalone executable or in an interpreted version that can be modified and run within Matlab (version 2019.b or later). It works with the appropriate Image Processing and Statistics and Machine Learning Toolboxes and can be edited with any text editor.

### Characterization of MSCs Differentiation by RT-qPCR

Following adipogenic induction, MSCs were harvested and lysed, and total RNA was isolated using the RNeasy Micro Kit (Qiagen). Complementary DNA (cDNA) synthesis was subsequently carried out with the Quantitect Reverse Transcription Kit (Qiagen), in accordance with the supplier’s protocol. Quantitative real-time PCR analysis was then performed as previously described [20]. Primer sequences are provided in Table 2. Gene expression levels were normalized to TBP (TATA-box binding protein), which served as the internal reference gene (Table 5).

**Table 5:** Primer’s sequence

Gene	Upper primer (5’ to 3’)	Lower primer (5’ to 3’)
TBP	TGCACAGGAGCCAAGAGTGAA	CACATCACAGCTCCCCACCA
CD44	CTTTCAATAGCACCTTGCCCAC	CCCTTCTATGAACCCATACCTGC
CEBPA	GCTGACCAGTGACAATGACCGCCT	GCCCCGCAGCGTGTCCAGT
CD90	GAACGTCACAGTGCTCAGAGACAAAC	TGTTCTGAGCCAGCAGGCTGA
ADIPOQ	CAGGCCGTGATGGCAGAGAT	CCCTTAGGACCAATAAGACCTGGAT
S100A4	CTCGGGCAAAGAGGGTGACAA	GCTTCATCTGTCCTTTTCCCCAA
FABP4	GGAAAGTCAAGAGCACCATAACCT	CTTTCATGACGCATTCCACCA

### Statistical Analysis

All experiments were performed using at least three independent biological replicates, with each experimental condition tested in duplicate or greater. Statistical analyses were conducted using non-parametric tests, including the Mann–Whitney U test for comparisons between two groups and the Kruskal–Wallis test for comparisons among multiple groups. Data are presented as mean ± standard deviation (SD). Statistical significance was defined as a two-sided p-value < 0.05. The numerical quantification of red pixels was evaluated and formatted using GraphPad PRISM 8 software (GraphPad Software Inc., San Diego, CA, USA).

## Results

### Literature Review

#### Composition of Heterogeneous Differentiation Media and Challenges in Standardization

An adipogenic differentiation protocol was proposed in 1999, involving a differentiation medium containing a cocktail of adipogenic inducers [21]. However, ten years later, data regarding the composition of adipogenic differentiation media remain limited and poorly standardized. Reproducibility between studies is considered low, with most protocols being internally developed by individual research teams [22]. In 2011, researchers noted this heterogeneity [14], particularly regarding variable concentrations of indomethacin (INDO), insulin, dexamethasone (DEX), and isobutyl methylxanthine (IBMX). They conducted a comprehensive review of adipogenic differentiation methods using BM-MSCs and ADSCs. For BM-MSCs, the cocktail could include 175 nM DEX and 50 mM INDO. Although some teams have demonstrated the efficacy of 170 nM insulin [23], this hormone is not mentioned because certain teams exclude it from their studies. The adipogenic cocktail differs for ADSCs, with the review suggesting a composition of 393 nM insulin and 100 nM DEX. In both cases, the medium should contain 10% FBS. The addition of 1 mM rosiglitazone is suggested [14], but the authors do not recommend the use of IBMX, despite its inclusion in some referenced studies. Furthermore, this review does not address adipogenic differentiation from WJ-MSCs or MSCs from other tissue sources, nor does it discuss the glucose concentration in the differentiation medium. Nevertheless, glucose concentration is subject to optimization. For BM-MSCs, it has been shown that a medium containing 1 g/L D-glucose is sufficient for adipogenic phenotype expression [23]. Beyond the differentiation media composition, variations in protocols across research teams and studies further complicate the comparability of results.

#### Heterogeneity in Protocol Design

The literature revealed heterogeneity in two independent methodological parameters: the adipogenic induction strategy and the duration of differentiation. Regarding the induction strategy, two principal approaches are commonly used. The first consists of alternating induction and maintenance phases, in which an induction medium enriched with adipogenic factors is periodically replaced by a maintenance medium depleted of several inducers [9, 10, 23–25]. The second consists of continuous adipogenic induction, in which the same differentiation medium is maintained throughout the experiment and renewed every two to three days [6, 12, 13, 21, 22]. Independently of the induction strategy, differentiation duration also varies considerably among studies. Across the 41 publications included in this review, differentiation periods ranged from 14 days to five weeks regardless of MSCs tissue origin. Although the median duration was 21 days, protocols using similar MSCs sources and comparable experimental designs differed by as much as two-fold in total differentiation time, illustrating the substantial methodological heterogeneity currently present in the field.

#### Heterogeneity and Limitations of Analytical Methods Used

The analytical procedures employed to report results are also heterogeneous, despite a significant trend favoring cytochemical staining techniques. For instance, results can be analyzed by staining lipid droplets formed in the cytoplasm using a 3% ORO emulsion after fixing cells with 4% paraformaldehyde (PFA). However, numerous other analytical approaches are also available. The ORO staining method works particularly well and allows for unequivocal visualization of newly formed lipid droplets. The stain can subsequently be solubilized in isopropanol and quantified using a spectrophotometric method, with a wavelength range of 490 nm to 510 nm. However, this method provides no information on the number of differentiated cells or the average lipid content per cell. Additionally, sample absorbance is subject to variations caused by the evaporation of isopropanol over time (as isopropanol is a volatile organic solvent). An enzymatic method provides an alternative, enabling the evaluation of triglyceride (Tg) content after extraction with DMSO. Tg levels are quantified using enzymatic kits and absorbance measurements [26]. Although this method is based on a chemically distinct principle from staining, it produces equivalent results and has limitations comparable to those of the ORO staining technique. To improve the reliability of these results, ORO staining can be analyzed using other analytical methods. To complement the spectrophotometric method, a semi-quantitative approach based on photographs of stained cells can also be employed. The ORO staining is visible under optical microscopy, allowing for the photography of relevant fields. These photos can then be used to quantify differentiation. One method involves a scoring system where the operator assigns a rank based on the proportion of cytoplasm occupied by lipid droplets [24, 27]. However, this method lacks precision and reproducibility and seems outdated in the digital age. Fortunately, the use of computer tools is advancing, and software like ImageJ software (NIH, Bethesda, MD) can count stained cells while tallying the total number of cells in a photo. After image thresholding, data is expressed as integrated density, obtained by multiplying the average intensity of the light (or color) signal by the area (or pixel count) of the photographed zone. This quantifies the total signal intensity. Although this method is reliable and validated, it is time-consuming and difficult to automate, making data processing lengthy, especially with numerous photos. Despite these limitations, many studies rely solely on these cyto-chemical staining procedures, evaluated through both spectrophotometric and semi-quantitative methods. These techniques are relatively easy, cost-effective [4], and capable of objectively quantifying observable biological mechanisms. Although adipogenesis can be tracked by observing lipid droplet formation, this depends on gene expression induced by the adipogenic cocktail. Analyses of gene and protein expression in cells during adipogenic differentiation are highly relevant and precise. Key transcription factors for adipogenesis can be analyzed using real-time quantitative PCR (RT-qPCR) or RNA sequencing (RNAseq). Many studies are complemented by analyzing the gene expression of pluripotency markers such as Oct4 and Nanog [28], as well as adipogenic markers upregulated during the adipogenic induction of MSCs. These include fatty acid-binding protein 4 (FABP4), peroxisome proliferator-activated receptor gamma-2 (PPAR-γ), lipoprotein lipase (LPL) [29], ADIPOQ, CEPBα [16], and insulin-like growth factor-binding protein 2 (IGFBP-2) [30]. These proteins are particularly studied to elucidate the mechanisms of adipogenesis and to evaluate the adipogenic potential of MSCs, which can vary significantly depending on their tissue of origin.

#### The Adipogenic Potential of WJ-MSCs is Challenging to Evaluate

Many research groups applying adipogenic differentiation protocols originally developed for BM-MSCs or ADSCs have consistently reported a limited adipogenic response in WJ-MSCs [9, 14]. Rather than complete absence of differentiation, WJ-MSCs typically exhibit sparse intracellular lipid droplet formation, delayed adipogenic kinetics, reduced Oil Red O staining, and incomplete activation of canonical adipogenic transcription factors including PPARγ, CEBPα, ADIPOQ and PLIN1 [12, 29]. Even when differentiation is prolonged to 5–8 weeks, WJ-MSCs generally develop fewer and smaller lipid droplets than BM-MSCs cultured under identical conditions [9, 12, 13]. These observations have raised the question of whether the apparent adipogenic limitation of WJ-MSCs reflects intrinsic biological properties or, alternatively, suboptimal differentiation conditions.

#### Composition of the Adipogenic Cocktail

Among the 41 analyzed studies, 11 employed commercial media such as StemPro™ (Gibco/Invitrogen) or StemMACS™ (Miltenyi), predominantly with MSCs derived from foetal tissues. Notably, 8 of these studies involved WJ-MSCs or umbilical cord MSCs (UC-MSCs), representing half of the studies focusing on these cell types. The remaining 30 studies detailed the composition of the differentiation medium, highlighting a consistent use of key components. IBMX (0.5 mM) was included in 28 studies with no variation in concentration. Dexamethasone (DEX) was also common, though concentrations varied, with a median value of 0.001 mM across 25 studies. Indomethacin (INDO) was used in 26 studies, with a median concentration of 0.1 mM, double the average dose reported in 2010. While INDO concentrations varied from 0.05 to 0.2 mM, they remained within a narrow range. Insulin was another universal component, with a median concentration of 10 µg/mL (1.72 mM), though three studies employed a lower dose of 5 µg/mL (0.86 mM). Together, IBMX, INDO, DEX, and insulin form the core of adipogenic differentiation cocktails. Additionally, 6 studies incorporated rosiglitazone (1–10 µM), with some exploring its adipogenesis-promoting effects in the absence of IBMX and INDO [10]. Hydrocortisone, included in 4 studies at a median concentration of 0.01 µM, was used alongside DEX rather than as a substitute. This data highlights the balance between standardization and flexibility in adipogenic cocktail formulations used in MSCs research. Building on these insights, we conducted an experimental comparison of the adipogenic differentiation potential of BM-MSCs and WJ-MSCs in various adipogenic differentiation media, with the aim of identifying the optimal standardization conditions in terms of duration and differentiation parameters.

### Classical Adipogenic Differentiation of Human MSCs Derived From Bone Marrow and Wharton’s Jelly

**Fig. 1:**
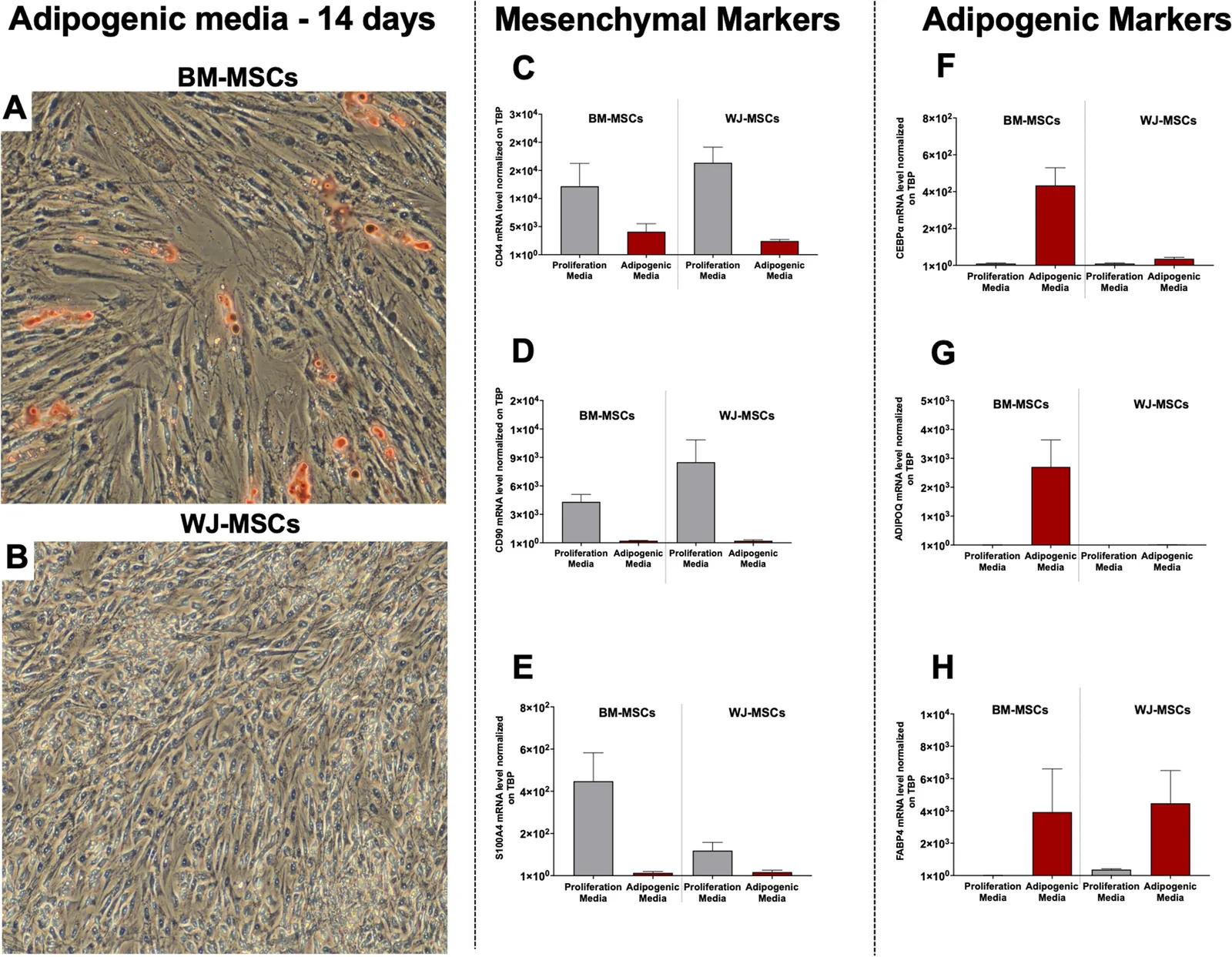
Distinct adipogenic differentiation capacities and transcriptional responses of BM-MSCs and WJ-MSCs under standard induction conditions. BM-MSCs and WJ-MSCs were cultured for 14 days under standard adipogenic induction conditions (LGI-). Representative Oil Red O (ORO) staining images show robust intracellular lipid accumulation in BM-MSCs (**A**), whereas WJ-MSCs failed to develop detectable adipogenic morphology under identical conditions (**B**). Expression of mesenchymal markers CD44 (**C**), CD90 (**D**), and S100A4 (**E**) was quantified in proliferative conditions (grey) and following adipogenic induction (red). Expression of adipogenic markers CEBP-α (**F**), ADIPOQ (**G**), and FABP4 (**H**) was similarly evaluated. BM-MSCs exhibited marked induction of canonical adipogenic transcriptional programs, whereas WJ-MSCs retained an incomplete adipogenic phenotype despite exposure to differentiation media. Data are presented as mean ± SEM

Adipogenic differentiation was evaluated in BM-MSCs and WJ-MSCs after 14 days of culture using Oil Red O (ORO) staining to detect intracellular lipid accumulation. BM-MSCs maintained in proliferation medium and used as negative controls did not exhibit detectable lipid droplets, confirming the absence of spontaneous adipogenic differentiation under these conditions. In contrast, BM-MSCs cultured for 14 days in standard adipogenic differentiation medium (LGI-) displayed a pronounced accumulation of cytoplasmic lipid droplets, as evidenced by intense Oil Red O staining, indicating efficient adipogenic differentiation (Fig. 1A). Similarly, WJ-MSCs cultured in proliferation medium for 14 days showed no evidence of lipid droplet formation despite marked cellular over confluence. However, unlike BM-MSCs, WJ-MSCs cultured in LGI- medium for 14 days failed to exhibit detectable lipid accumulation following Oil Red O staining, suggesting a limited or absent adipogenic differentiation capacity under these experimental conditions (Fig. 1B**).** Under these standard conditions, the analysis of gene expression associated with mesenchymal, and adipogenic phenotypes revealed several notable findings. First, the mesenchymal phenotype was characterized by the expression of markers such as CD44 (Fig. 1C), CD90 (Fig. 1D), and S100A4 (Fig. 1E). Both BM-MSCs and WJ-MSCs exhibited similarly high expression levels of these mesenchymal markers when cultured in standard proliferation medium. As expected, culture in adipogenic differentiation medium resulted in a marked downregulation of these mesenchymal markers, consistent with a phenotypic switch associated with lineage commitment. Next, the expression of adipogenic markers, including CEBP-α (Fig. 1F), ADIPOQ (Fig. 1G), and FABP4 (Fig. 1H) was assessed. As anticipated, none of these adipogenic genes were expressed in either BM-MSCs or WJ-MSCs cultured in proliferation medium. Upon exposure to adipogenic differentiation conditions, only BM-MSCs demonstrated substantial induction of adipogenesis-related genes. In contrast, WJ-MSCs did not express CEBP-α or ADIPOQ, while FABP4 expression was detected at levels comparable to those observed in BM-MSCs.

### Adipogenic-optimized Media Enhance MSCs Adipogenic Differentiation After 14 Days

**Fig. 2:**
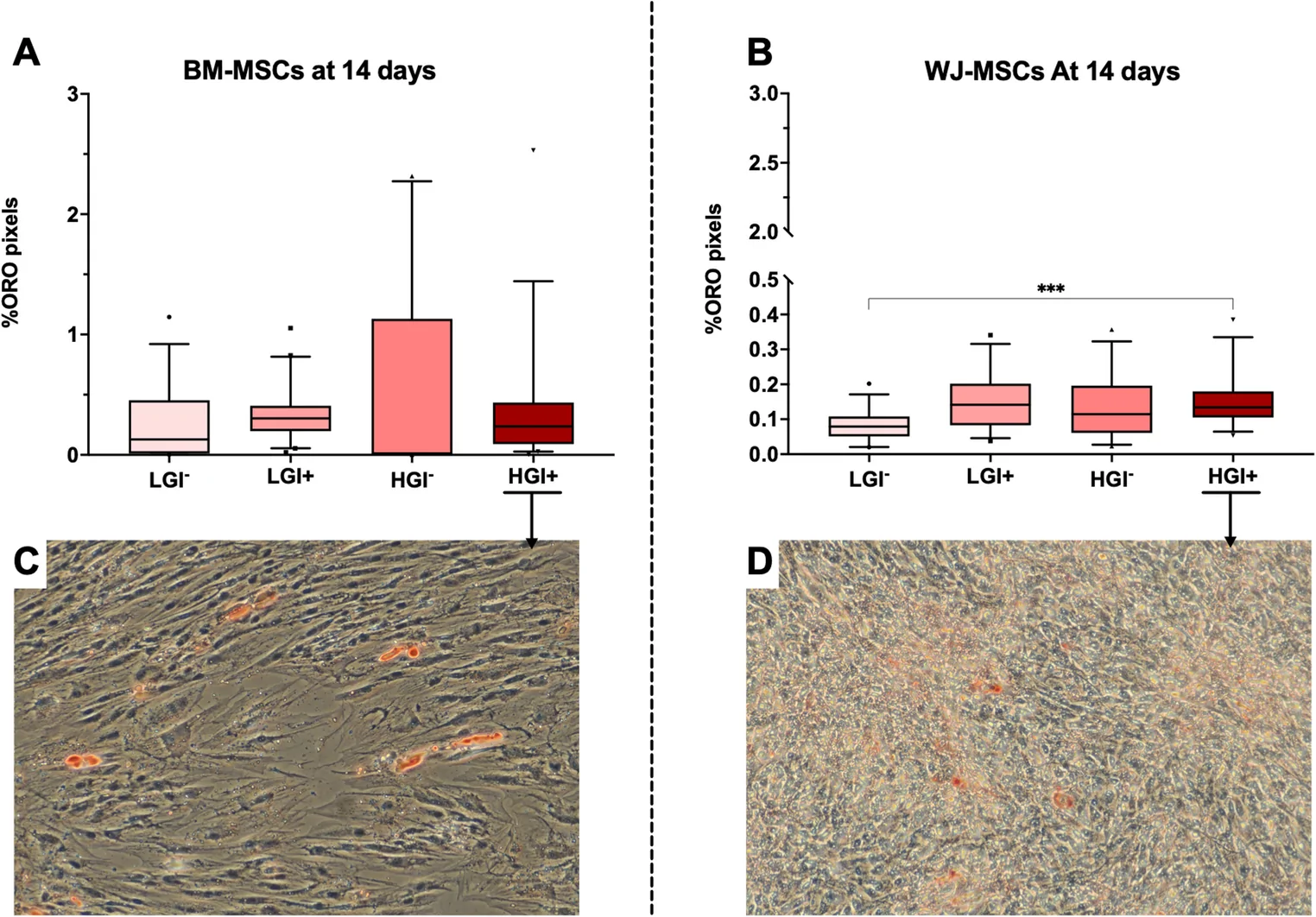
Metabolic optimization partially enhances adipogenic differentiation after 14 days of induction. Adipogenic differentiation of BM-MSCs and WJ-MSCs was evaluated after 14 days under four adipogenic conditions differing in glucose availability and insulin supplementation. LGI-: standard adipogenic medium containing low glucose (1 g/L) without insulin; LGI+: low glucose supplemented with insulin (10 µg/mL); HGI-: high-glucose medium (4.5 g/L) without insulin; HGI+: high-glucose medium supplemented with insulin (10 µg/mL). Quantification of Oil Red O staining revealed that metabolic supplementation modestly increased adipogenic differentiation, particularly under combined high-glucose and insulin conditions. Data are presented as median with 5th–95th percentiles

To assess the relevance of glucose and insulin supplementation, three additional optimized adipogenic media were tested as described in the methods section. Accordingly, BM-MSCs and WJ-MSCs were exposed to four distinct adipogenic conditions during 14 days of adipogenic differentiation. The optimization of the differentiation medium appears to have only a limited effect on BM-MSCs (Fig. 2A). Indeed, an increasing trend is observed when the medium is supplemented with glucose (HGI-) or with glucose and insulin (HGI+); however, this increase does not reach statistical significance. Moreover, the phenotype of BM-MSCs obtained under these conditions (Fig. 2C) does not appear to differ from that observed with the standard medium (LGI-) (Fig. 1A). A similar upward trend is also observed in WJ-MSCs (Fig. 2B). Although the quantitative values remain low, adipogenic differentiation is detected when the medium is supplemented with both glucose and insulin (HGI+). Under these conditions, the modest formation of lipid droplets is observed within the cytoplasm of WJ-MSCs (Fig. 2D). Furthermore, the expression of the adipogenic phenotype, while still limited, is significantly higher than that measured in WJ-MSCs cultured in standard adipogenic medium (Figs. 1B and 2B).

### Optimized Glucose and Insulin Conditions Promote Adipogenic Differentiation of WJ-MSCs Over 30 Days

**Fig. 3:**
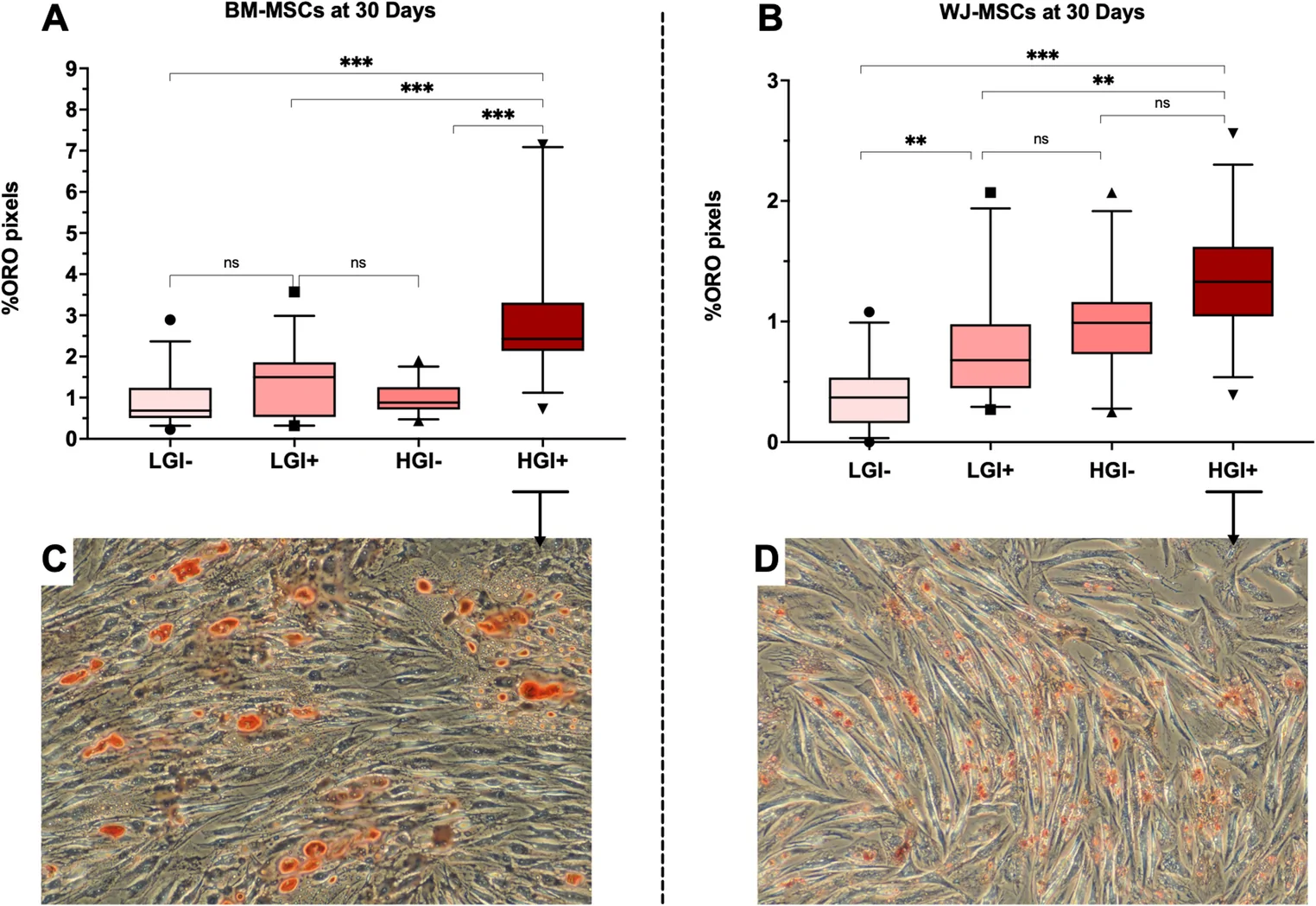
Glucose and insulin synergistically promote adipogenic commitment in BM-MSCs and WJ-MSCs. BM-MSCs and WJ-MSCs were cultured for 30 days under four adipogenic conditions combining differential glucose concentrations and insulin supplementation. LGI-: standard adipogenic medium containing low glucose (1 g/L) without insulin; LGI+: low glucose supplemented with insulin (10 µg/mL); HGI-: high-glucose medium (4.5 g/L) without insulin; HGI+: high-glucose medium supplemented with insulin (10 µg/mL). Quantitative image analysis of Oil Red O staining demonstrated that combined metabolic enrichment with high glucose and insulin significantly enhanced adipogenic differentiation in both MSC populations, with the strongest effect observed in WJ-MSCs under prolonged induction conditions. Data are presented as median with 5th–95th percentiles

In BM-MSCs, the effect of differentiation medium optimization contrasts markedly with that observed in WJ-MSCs. Under standard adipogenic conditions (LGI-), BM-MSCs already exhibited clear and unequivocal adipogenic differentiation, characterized by readily visible lipid accumulation. Neither insulin supplementation alone (LGI+) nor increased glucose concentration alone (HGI-) significantly enhanced adipogenic differentiation compared with the standard condition. In contrast, culture in the fully optimized HGI+ medium (high glucose, 4.5 g/L, combined with an insulin-enriched adipogenic cocktail, 10 µg/mL) resulted in a significant increase in adipogenic differentiation relative to LGI- (*p* < 0.0001), as well as compared with the two other optimized conditions (*p* < 0.0001) (Fig. 3A). These results indicate that BM-MSCs possess a strong intrinsic adipogenic capacity that is already efficiently induced under standard conditions, with only limited additional benefit from medium optimization unless glucose and insulin are combined. In WJ-MSCs, adipogenic differentiation remained substantially lower and required prolonged induction to become detectable. After 30 days of differentiation, the standard adipogenic medium (LGI-) was still insufficient to induce robust adipogenesis. However, supplementation of the adipogenic cocktail with insulin (LGI+) significantly enhanced differentiation (*p* = 0.0038). Similarly, increasing glucose concentration alone (HGI-) markedly improved adipogenic commitment compared with the standard condition (*p* < 0.0001). The highest level of differentiation was achieved in the optimized HGI+ medium, which significantly increased adipogenesis relative to LGI- (*p* < 0.0001) (Fig. 3B). Collectively, these findings demonstrate that the combined supplementation of glucose and insulin reveals the intrinsic adipogenic potential of WJ-MSCs in vitro and that a minimum induction period of 30 days is required for this phenotype to become fully apparent (Fig. 3D).

## Discussion

We demonstrated here that adipogenic commitment of human MSCs is profoundly influenced by both tissue origin and metabolic context. To our knowledge, this is the first study combining a structured review of adipogenic protocols with experimental optimization of glucose and insulin conditions to establish standardized recommendations for WJ-MSCs adipogenesis. Consistent with previous reports, BM-MSCs displayed robust adipogenic differentiation under conventional induction conditions, whereas WJ-MSCs remained largely refractory to standard adipogenic media [11, 31]. However, our findings further show that this apparent adipogenic restriction of WJ-MSCs is not absolute. Instead, prolonged exposure to optimized metabolic conditions combining elevated glucose and insulin supplementation progressively unmasked a substantial adipogenic potential in these cells [15, 16]. These results suggest that the limited adipogenesis classically reported in WJ-MSCs may primarily reflect inadequate induction conditions rather than an intrinsic incapacity to engage the adipogenic program. Importantly, our study highlights the major impact of metabolic environment on MSCs fate specification. While adipogenic differentiation protocols are widely used across the field, they remain highly heterogeneous regarding glucose concentration, insulin supplementation, induction duration, and analytical procedures [14]. Such variability likely contributes to the inconsistent characterization of MSCs adipogenic potential reported between studies and across tissue sources [31]. By integrating a systematic literature analysis with experimental validation, our work identifies glucose availability and insulin signaling as key determinants of adipogenic commitment, particularly in perinatal MSCs populations [16].

Glucose is not only an energy substrate but also a critical regulator of adipogenic differentiation [32]. During lineage commitment, MSCs undergo a metabolic shift toward increased glycolytic activity to meet the energetic and biosynthetic demands required for lipid synthesis. Glycolytic intermediates provide precursors for de novo lipogenesis, while glucose-derived acetyl-CoA and glycerol-3-phosphate are essential for triglyceride production and lipid droplet formation. Consequently, increased glucose availability supports both the metabolic and anabolic processes required for activation of the adipogenic program and maturation of adipocytes [16]. In parallel with providing metabolic substrates, adipogenic differentiation also requires activation of insulin-dependent signaling pathways. Indeed, Insulin acts as a master regulator of adipogenesis by coordinating metabolic activity with transcriptional differentiation programs [32, 33]. Binding of insulin to its receptor activates the PI3K/AKT signaling pathway, which promotes glucose uptake and stimulates the expression and activity of the master adipogenic transcription factor PPARγ. Activation of AKT also enhances C/EBPα expression and suppresses anti-adipogenic signaling pathways, thereby reinforcing adipocyte commitment and terminal differentiation. The synergistic effect observed in our study under combined high-glucose and insulin conditions is therefore consistent with the established role of insulin signaling in promoting adipogenic differentiation [30]. An additional explanation for the differential adipogenic response lies in the developmental origin of the cells. WJ-MSCs are fetal-derived cells and retain metabolic characteristics distinct from those of adult BM-MSCs. This fetal metabolic program may require stronger or more prolonged metabolic stimulation to activate adipogenic differentiation, providing a biological explanation for the delayed response of WJ-MSCs observed in our study and in previous reports.

Our data further reinforce the concept that MSCs retain a strong epigenetic and metabolic memory of their tissue of origin [34]. BM-MSCs, which physiologically reside within an adipose-rich bone marrow niche, rapidly activated canonical adipogenic pathways and accumulated intracellular lipids under standard induction conditions [35]. In contrast, WJ-MSCs required prolonged metabolic stimulation before detectable adipogenesis emerged, supporting the idea that perinatal MSCs exist in a more primitive developmental state associated with distinct metabolic requirements and delayed lineage commitment kinetics [29]. Notably, optimized conditions induced not only lipid accumulation but also partial activation of adipogenic transcriptional markers in WJ-MSCs, indicating engagement of a bona fide differentiation program rather than nonspecific lipid storage [30].

This literature review identified a baseline composition for the differentiation medium and adipogenic cocktail, as well as an induction strategy used in in vitro studies involving MSCs from various sources. However, the lack of standardization prevents the establishment of a clear consensus on the complete composition of the adipogenic differentiation medium, particularly for WJ-MSCs. Although commercial adipogenic differentiation kits are available [5, 30, 34, 36–42], it is crucial to determine a composition for the adipogenic differentiation of WJ-MSCs, and thus assess the necessity of insulin supplementation at a concentration of 10 µg/mL and the glucose concentration. Initially, the studies conducted in the 2000s [11, 13, 24, 37] where no adipogenic differentiation was obtained from MSCs derived from the umbilical cord, in contrast to that observed from BM-MSCs and ADSCs. In the 2010s, experimental protocols revealed the adipogenic potential of WJ-MSCs, though they never achieved differentiation comparable to that observed in BM-MSCs [29, 36, 38–40, 43]. Across the literature, WJ-MSCs are consistently reported to remain at a relatively early, less-developed stage of differentiation by the conclusion of induction. They display few lipid vacuoles, with small lipid droplets that are heterogeneously distributed within the same culture [38]. For instance, Chang et al. [36] quantified the proportion of adipogenic induced WJ-MSCs as being below 5% of the cell culture, even after five weeks of induction. In contrast, BM-MSCs exhibit lipid droplets in 80% of cells within just three weeks of induction. Lipid droplets appeared in their cytoplasm as early as the first week, reaching over 80% of the cells by week 3.

By combining a structured literature review with experimental validation, our study highlights the absence of consensus regarding adipogenic induction protocols and identifies key parameters, particularly glucose concentration and insulin supplementation, as critical determinants of differentiation efficiency. Importantly, our results show that, although WJ-MSCs have long been considered poorly adipogenic, their differentiation potential can be partially revealed under optimized conditions, suggesting that previously reported limitations may be, at least in part, methodological rather than intrinsic. Building on the impact of the cellular microenvironment on differentiation, recent studies [41, 42] have gone further by proposing the enrichment of the medium with energetic substrates such as exogenous fatty acids. Their hypothesis is based on the analysis of the lipidomic profile, which differs radically depending on the cell type studied. Wu et al. proposed that the triglyceride composition of WJ-MSCs imposes a kinetic constraint on adipogenesis, as intracellular lipid stores must progressively accumulate before stable lipid droplet formation can occur. This would result in a slower differentiation rate and a lower adipogenic differentiation efficiency, than that observed in MSCs derived from adipose tissue, for example [44]. The emerging concept of lipidomic memory proposes that MSCs retain characteristic lipid signatures from their source tissue, and that these molecular features may contribute to their specific differentiation potential and immunomodulatory properties [45–47].

Several limitations of this study should be acknowledged. First, our comparative analyses focused primarily on WJ-MSCs and BM-MSCs. Although BM-MSCs constitute one of the most extensively characterized MSCs populations and remain a reference standard for trilineage differentiation studies, inclusion of adipose-derived stromal/stem cells (ADSCs) would have provided an additional and highly relevant benchmark for adipogenic commitment, given their intrinsically robust adipogenic capacity [48]. Future studies integrating ADSCs under identical metabolic and temporal conditions would therefore help refine the hierarchy of adipogenic competence across MSCs sources and further clarify tissue-specific differentiation constraints. Despite this limitation, our findings clearly demonstrate that the reduced adipogenic response commonly attributed to WJ-MSCs is highly dependent on induction conditions and does not necessarily reflect an intrinsic inability to engage adipogenic programs. Rather, the marked discrepancies observed between studies likely arise from the absence of standardized differentiation procedures across MSCs subtypes. This issue is particularly critical in the MSCs field, where substantial methodological heterogeneity persists regarding glucose concentration, insulin supplementation, induction duration, passage number, and analytical endpoints. Such variability severely complicates inter-study comparisons and contributes to inconsistent conclusions regarding the true adipogenic potential of perinatal MSCs. Based on both our experimental observations and systematic analysis of the literature, we propose a harmonized adipogenic differentiation framework applicable to both BM-MSCs and WJ-MSCs. Optimal differentiation was achieved using early-passage cells (preferably ≤ P5) seeded at approximately 80% confluence prior to induction. The differentiation medium consisted of high-glucose DMEM (4.5 g/L glucose) supplemented with 10% fetal bovine serum and 1% antibiotics, together with a classical adipogenic cocktail containing dexamethasone (10^-6 M), indomethacin (60 µM), 3-isobutyl-1-methylxanthine (0.5 mM), and recombinant human insulin (10 µg/mL). Importantly, our data indicate that prolonged induction is required to reveal the adipogenic competence of WJ-MSCs, with robust lipid accumulation becoming detectable only after extended culture periods. We therefore recommend maintaining induction for up to 30 days with medium renewal every 72 h. Finally, quantitative assessment of adipogenesis remains another major source of variability across studies. In this context, the Fiber-ML platform proved to be a robust, accessible, and reproducible tool for lipid droplet quantification, enabling standardized comparative analysis between MSCs populations [18]. The implementation of harmonized culture conditions together with objective image-based quantification strategies may contribute substantially to improving reproducibility and comparability within the MSCs adipogenesis field.

All in all, our findings demonstrate that adipogenic commitment of MSCs is strongly influenced by the metabolic microenvironment. While BM-MSCs readily undergo adipogenesis under standard conditions, WJ-MSCs require prolonged exposure to optimized metabolic cues, particularly elevated glucose and insulin, to reveal their adipogenic potential. These results challenge the prevailing view that WJ-MSCs are intrinsically poorly adipogenic and instead support a model in which adipogenic competence depends on appropriate induction conditions and differentiation kinetics. Importantly, our findings support the establishment of standardized adipogenic differentiation protocols for MSCs from different tissue sources, thereby improving reproducibility, inter-study comparability, and quality control for MSCs characterization in regenerative medicine.

## Data Availability

No datasets were generated or analysed during the current study.

## Abbreviations

ADSCs: Adipose-derived Stromal/Stem cells
BM-MSCs: Bone Marrow Mesenchymal Stromal/Stem Cells
DEX: Dexamethasone
FIBER-ML: FIBER Machine learning
FBS: Fetal Bovine Serum
IBMX: isobutyl methylxanthine
INDO: Indomethacin
ISCT: International Society for Cell and Gene Therapy
MSCs: Mesenchymal Stromal/Stem Cells
ORO: Oil Red O
UC: umbilical cord
WJ-MSCs: Wharton Jelly derived Mesenchymal Stromal/Stem Cells
